# Prevalence, student nurses’ knowledge and practices of needle stick injuries during clinical training: a cross-sectional survey

**DOI:** 10.1186/s12912-021-00711-2

**Published:** 2021-10-04

**Authors:** Mohammad Al Qadire, Cherry Ann C. Ballad, Omar Al Omari, Khaldoun M. Aldiabat, Yousef Abu Shindi, Atika Khalaf

**Affiliations:** 1 College of Nursing, Sultan Qaboos University, P.O. Box 66, PC 123 Muscat, Sultanate of Oman; 2 Faculty of Nursing, Al Al-Bayt University, P.O. Box 130040, Mafraq, 25113 Jordan; 3 Psychology Department, Sultan Qaboos University, PC 123 Muscat, Sultanate of Oman; 4 Faculty of Health Sciences, Kristianstad University, SE: 291 88, Kristianstad, Sweden

**Keywords:** Needles, Infection control, Students, Practice, Oman

## Abstract

**Background:**

The incidence of needle stick injuries is higher among nurses with a low level of knowledge on the prevention of needle stick injury, and who have not received the relevant training during their undergraduate study. The aim of this study was to determine the level of knowledge of the prevention guidelines and the prevalence of needle stick injury among students in Oman.

**Methods:**

An online cross-sectional survey using a questionnaire and involving 167 students from a governmental university was conducted. The questionnaire consists of 30 questions; eight general questions, knowledge related questions, and questions about risk factors, prevention measures, and actions in a case of needle stick injury. Ethical approval was obtained and the link to the survey was shared with students using their university email portal.

**Results:**

Of the participants, 81.2% were females; mean age was 23.3 (SD = 4.5) years. The mean total knowledge score was 6.6 out of 10 (SD = 2.1). In addition, 18.2% (*n* = 32) of the students experienced needle stick injury. Most of the injuries 71.9% (*n* = 24) occurred during medication preparation and administration. The main cause of NSI as reported by students was recapping the needles (59%, *n* = 19).

**Conclusions:**

This study demonstrates that student nurses have a moderate level of knowledge about needle stick injury prevention measures and lack many facets of safe infection control practice. These findings require the collaborative effort of nursing administrators from both academic and clinical areas, to develop effective strategies to reduce or eliminate the occurrence of needle stick injury.

## Background

Needle stick injury (NSI) is a nonintentional penetration of the skin by a needle [1]. Several studies revealed that nurses among all other health care workers are most predominantly affected primarily due to the nature of their job [2–7]. Interestingly, the incidence of NSI is higher among nurses who had low level of knowledge on the prevention of NSI and did not receive the relevant training or education which they mostly gained during their undergrdaute study [8, 9].

Multitude of studies worldwide reveal varying degree of prevalence of NSI among nursing students (from 11.8 to 85.0% [10–21]). Alarmingly, majority of these studies indicated underreporting of NSI incidents (ranging from 13.1 to 62.1% [10–21]). Most of the NSI incidents occurred during drug administration, drug preparation, recapping, carrying syringes without proper receptacle, improper needle disposal, opening needle cap, sudden movement of patient during injection, blood sampling, and suturing [10–14, 21–23]. Higher incidence is noted among students who do not wear proper protective equipment [12–14, 16].

In terms of demographic characteristics, several studies had divergent results, for example one study reported that NSI occurred more frequently among female students [14], or male students [12], others found it equal in both genders [15]. Positive correlation of NSI with year of study was noted [11, 12] whereas other studies reported a negative correlation [10, 13, 17]. Remarkably, NSI is higher among students who did not receive proper safety or work-related training [11, 12, 14], inadequate knowledge, skills and experience [12, 13, 15], and who were inattentive or distracted during the procedure [13, 15]. More frequent NSI also is noted when students were not supervised by their instructors [12].

While an ample portion of NSI were either on uncontaminated needles or unknown patient serological status, it is worrisome to note that considerable percentage of contaminated needles were positive for HIV, Hepatitis B and Hepatitis C [10, 12, 15]. Despite of this alarming data, majority of students were reluctant to undergo post-exposure diagnosis or prophylaxis. Roughly, around 10 to 85% of students who had NSI went for diagnostic test [10, 13, 18, 21] and students who sought prophylactic treatment ranges from none to 58% [12, 15–19, 21]. Moreover, studies document that not all students completed their vaccination prior to clinical exposure [17, 19, 21]. While numerous studies were conducted in various countries, this topic remains unexplored in the region (only one study in the Arab region). This study therefore aims to determine the level of knowledge and the prevalence of NSI among Omani nursing students.

## Aims

The current study aimed to determine the prevalence of needle stick injuries during clinical training among Omani nurse students and to assess their knowledge of the prevention guidelines for NSI as well as examine the associated demographical characteristics with the level of knowledge.

## Methods

### Design

A descriptive cross-sectional survey design was used.

### Sample and sample size

The sample was nurse students attending a governmental university in Oman. All students were invited to take part in this study as long as they agreed on participation. First year students were excluded since they did not start their clinical training in hospitals yet and were therefore not at risk for needle stick injury. The knowledge and awareness about infection control measures develop further when students evolve in their studies and prepare for the clinical practice in hospitals. Consecutive sampling approach was adopted.

#### Sample size

The sample size depended on the distribution of students’ responses on the needle stick injury knowledge questionnaire. Based on the literature, 60% correct answers rate is expected. The faculty of nursing have 300 students (excluding first year students), the required sample size is 166 students (calculated from: http://www.raosoft.com/samplesize.html). This would allow to estimate the level of student knowledge regrading NSI with 95% and ± 5% margin error.

### Settings

This survey was conducted on nurses’ students within one university which is a governmental university. Nursing college within this university has around 400 students (including first-year students). This study utilized an online survey form, the link of the survey combined with an invitation letter were sent to the students through students’ email portal.

*Instruments*The instrument that was developed by Suliman et al. (2018) [18] was used to collect data in this study. The tool consists of 30 questions divided into three parts. The first contains eight general questions about age, gender, mood of admission, university, year of study, GPA, number of Hepatitis B vaccine doses, previous training of NSI prevention, and awareness of the universal standard precautions. The second is the knowledge part, it contains four True/ False questions and six multiple-choice questions about risk factors, prevention measures, and actions in case of NSI occurred. To avoid guessing, the “I don’t know” choice was added. Each correct answer assigned a score of 1 and zero for other choices. A total score with range from 0 to 10 can then be calculated. The third part comprises 11 questions about experienced occurrence of NSI, frequency, reporting of NSI, reason for not reporting and follow up procedures. Content validity of the tool was established.

### Procedure

The researchers obtained ethical approval from the university. Then, a pre prepared invitation letter combined with the link for the online questionnaire were sent for all students (except first year students). The students were instructed to read the invitation letter, orient themselves with study purpose and requirements. And thereafter if they agreed on participation, they had to click on the Link for the survey. Students were informed that they have to click submit once they finish. Also, they were informed that they have the chance to edit or even delete their response to the questionnaire. Three reminders were sent every 2 weeks. Data collection was commenced in the period between July to September 2020.

### Ethical considerations

Ethical approval from the ethics committees was obtained from College of Nursing, Sultan Qaboos University (Ref. No. CON/NF/2020/27) prior to data collection. All students were given the right to participate or not. No identifying information were requested or recorded. Further, to avoid coercion, no direct contact between the members of the research team and students have occurred. A third-party person, from the admission office, took the responsibility of sending emails to students. Informed consent was secured through information on the first page of the survey in which students were informed that by pressing the “continue” bottom they gave their consent to participate in the study.

### Data analysis

Data entry and analysis was conducted using SPSS version 21. Descriptive statistics such as mean, standard deviation, frequency, and percentages were used to summarize sample characteristics and responses on the questionnaire. Unpaire t-test and ANOVA were used to test the difference in mean knowledge score with regard to selected stduents demographics.

## Results

### Sample characteristics

A total 167 students completed the questionnaire, of them 81.2% were females with mean age of 23.3 (SD = 4.5) years. About 43% of participants were in their fifth year within the regular bachelor nursing programme (76.1%). Only 25% of the students were given the three and booster doses of hepatitis B vaccine. Table 1 details sample characteristics.

**Table 1:** Demographic characteristics of nursing students (*n* = 167)

Characteristic	Frequency (%)	Mean (SD)
**Age (year)**		23.3 (4.5)
**Grade Point Average (4-point system)**		2.9 (0.5)
**Gender**		
Male	33 (18.8)	
Female	143 (81.2)	
**Study year**		
Second year	18 (10.2)	
Third year	15 (8.5)	
Fourth year	67 (38.1)	
Fifth year	76 (43.2)	
**Type of Admission**		
Regular admission	143 (76.1)	
Bridging system	42 (23.9)	
**Have You Received Hepatitis B Vaccine?**		
None	25 (14.2)	
First does only	29 (16.5)	
First and second doses	50 (28.4)	
First, second, and third doses	28 (15.9)	
First, second, third, and poster doses	44 (25.0)	
**Previous education about proper handling of needles and sharp objects?**		
Yes	152 (86.4)	
No	24 (13.6)	

### Needle stick injuries

As many as18.2% (*n =* 32) of the students experienced NSI, 81% (*n* = 26) of them experienced the injury once while only six students (19%) had NSI twice or more. It was found that needles caused 84.2% (*n* = 27) of injuries and the rest (15.8%, *n =* 5) caused by blades. Students reported that 71.9% (*n =* 24) of injuries occurred during medication preparation and administration, 18.8% (*n* = 6) during blood extraction, and 9.3% (*n* = 3) during canulation. The main cause of NSI as reported by students were as following: recapping the needles (59%, *n* = 19) unexpected patients’ movement (28%, *n* = 9), and talking with others (13%, *n* = 4).

Once the injury occurred, 50.5% of students reported cleaning the site of injury with disinfectant solution. However, 23.8% squeezed the site of injury and 25.7% took no action. Only 40% of students got blood investigations after the injury. Finally, 53.1 reported the incidence to their instructor or infection control units in the training settings.

### Students’ knowledge of needle stick injuries

The mean of total knowledge score was 6.6 out of 10 (SD = 2.1). Table 2 shows the nurses correct answers. Most of students (90.9%) correctly identify the definition of NSI. In addition, 87.5% of students correctly knew that needle recapping is not recommended, and it is the most common cause of NSI. In contrast, only 30.1% of participants identified the required number of hepatitis B vaccine. Also, only 38.6% knew the correct action to be taken immediately after being subjected to NSI. Further details are shown in Table 2.

**Table 2:** Students Nurses Knowledge of Needle Stick Injuries (*n* = 176)

Item No.	Question	Correct Responses	
		n	%
1	Needle stick injury is defined as wounds caused by needles that accidentally puncture the skin. (True*)	160	90.9
2	Recap of the syringe after performing nursing interventions is recommended to decrease the risk of needle stick injury. (False*)	154	87.5
3	The most common cause of needle stick injury is during and after use of needles. (True*)	154	87.5
4	Which disease can be prevented by vaccine? (Hepatitis B*)	113	64.2
5	How many doses are required for full protection from hepatitis? (Three doses*)	53	30.1
6	What precautions are needed to avoid needle stick accidents? (Safer devices & technics and gloves*)	124	70.5
7	What are the blood-borne pathogens that medical staff are most commonly exposed to when they experience NSI? (Hepatitis B & C, HIV*)	122	69.3
8	What is the maximum capacity for a sharps’ container? (75%*)	72	40.9
9	After performing a procedure that involves a needle, which practice is recommended to decrease the risk of injury? (Dispose in a sharps container*)	145	82.4
10	Which action is recommended to decrease the risk of infection immediately after experiencing NSI? (Wash area with soap and water*)	68	38.6

*The correct answer

### Comparisons of knowledge Total score

To understand if there are differences in the mean of the total knowledge score with regard to participant characteristics, unpaired t-test and ANOVA were conducted. The t-test showed that there was no significant difference with regard to gender. However, bridging students have significantly higher mean of total knowledge score than regularly admitted students do. Further, participants who received previous education about NSI had significantly higher total knowledge score that those who did not (Table 3). Finally, ANOVA test showed that there was no significant difference in the mean of total knowledge score with regard to study year (*p* > .05).

**Table 3:** Comparisons of Needles Stick Injuries mean knowledge score and selected demographics (*n* = 167)

Characteristic	Mean (SD)	Test	Result (df)	***P*** value
**Gender**		t-test	0.58 (40.8)	.564
Male	6.4 (2.6)			
Female	6.7 (1.9)			
**Study year**		ANOVA	0.69 (3.0)	.554
Second year	6.3 (2.8)			
Third year	6.8 (1.1)			
Fourth year	6.4 (2.0)			
Fifth year	6.8 (2.1)			
**Type of Admission**		t-test	−3.8 (62.0)	*p* < .001*
Regular admission	6.3 (1.9)			
Bridging system	7.7 (2.2)			
**Previous education about proper handling of needles and sharp objects?**		t-test	−3.6 (29.1)	.001*
Yes	6.9 (2.2)			
No	5.1 (1.9)			

*Significant at *p* ≤ .05

## Discussion

### Prevalence of NSI and practice of prevention measures

The results of this study demonstrate that prevalence of at least one incident of NSI group was 18.2%. Lower prevalence was recorded in some previous studies [15, 21] and higher in some others [16, 18]. However, other studies recorded twice or thrice as much [10–12, 14, 19]. Moreover, 81% of the reported incident had it for the first time comparable to other studies [11, 16, 21]. Other studies recorded that around 50% had 2 to 3 or more incidents of NSI, which is relatively higher [10, 18]. In contrary, only a quarter of the students reported receiving the three doses and booster shot of Hepatitis B vaccine prior to their clinical experience. This is the lowest compared to the findings of other studies where vaccination ranged from 57 to 96% [17, 19, 21]. The setting where this study was conducted required complete vaccination prior to students’ clinical duty. Designated hospitals as training sites apply the same condition. Hence, this result is alarming and needs further verification; otherwise, nursing schools should adopt approaches to enhance students’ vaccination before clinical placement must be instituted.

Eighty-four percent of the NSI incidents were caused by needles which is similar though lower in other studies [14, 15]. Most of the NSI occurred during medication preparation and administration similar to other studies but lower in occurrence [10, 12, 16]. The high incidence of NSI during medication administration is not surprising since this is the most common nursing procedure being carried out on a daily basis that involves the use of sharps and needles [24]. Also, while students in this study had a considerable practice on medication administration in the laboratory prior to clinical placements, this does not guarantee seamless transition into actual clinical training as shown in one study. Rigorous follow through from theory to practice is mandatory.

Majority of the NSI occurred when the students recap the needles (59%). This is alarmingly higher compared to others studies where recapping is at a modest rate [15, 16, 21]. A WHO-International Council of Nurses (ICN) collaboration in 2004 identified recapping needles as one of the major causes of NSI [25]. Akin to this, ICN recommend not to recap needles to prevent NSI [25]. If it becomes extremely necessary or unavoidable, one-hand scoop technique must be used [25]. Clearly, the practice of recapping needles in this study requires stringent attention and correction.

Second cause of NSI in this study was the unexpected patients’ movement at 28%, similar with one study [12]. Third reported cause at 13% is being distracted or talking with others which is much lower compared to other studies where NSI occurred due to inattentiveness [13, 15]. Overall, several other facets must be factored in to understand NSI occurrence. Studies show that NSI is also related to inadequate clinical instructor to student ratio, unsatisfactory clinical and teaching competencies of clinical instructorss, students’ insufficient skills and lack of proper resources in the hospital [24]. These areas must be looked into in order to get a clear perspective and institute strategies aptly addressing such determinants.

More than half (53.1%) of the NSI incidents were reported by the students to the clinical instructor or infection control unit in their training sites. The literature denotes varying degree of reporting across countries. Though, NSI reporting in this study is only at a partial rate, this is more desirable compared to other studies that recorded very low reporting [14–17], except for two studies that had higher reporting rate [12, 13]. It is evident that underreporting is a vital area of concern in NSI. The need to encourage a proactive culture in addressing clinical problems and help students develop effective communication skills are fundamental to address this concern.

At the occurrence of NSI, 50.5% of the students reported cleaning the site of injury with disinfectant solution as their immediate corrective action. Such action is higher in one study [13], similar in another [12], but lower in one study [16]. A quarter of the students squeezed the site of injury similar to one study but only at minimal rate [12]. Disturbingly, a quarter of the students took no action at all comparable to other studies [12, 17]. Recommended post-exposure first aid care for NSI is to wash the affected area with soap and water and allow it to bleed freely [25]. Half of the nursing students performed proper care, but education and training need to be heightened to correct the practice of the other students and ensure appropriate post-NSI management.

Only 40% of the study group went for blood investigations after the injury. Other studies reported lower rate [13, 17, 18] except for one study where around three fourths had their serologic testing [21]. This finding significantly can put nursing students at risk in the development of blood-borne infections such as Hepatitis B and C and HIV-AIDS [22]. Without blood investigations to base future action, post-exposure prophylaxis (PEP) which includes vaccines and antiviral treatments are compromised. The longer the delay, the less effective the PEP will be. Education and support must be provided to students to ensure proper implementation of PEP protocol.

### Knowledge of NSI

Studies exploring nursing students’ knowledge on NSI are limited. In this study, students had moderate knowledge on NSI prevention (6.6 out of 10, SD = 2.1) comparable to previous works [18, 26]. Studies done on medical and dental students show moderate to high knowledge [27]; surprisingly, nurses and other healthcare workers’ knowledge on NSI was low [28, 29]. It is important to note that adherence to standard precautions is significantly related to level of knowledge [30]. The lower the knowledge, the poorer the adherence; hence, this may lead to greater NSI incidence. Long-term educational programs directed to improve nursing students’ knowledge is essential.

Some aspects where the students scored low in knowledge may predispose them to greater risk or unsafe practice. For example, only a third were able to identify the required doses for full protection from Hepatitis. Misperception of required vaccine dose and frequency affects the actual completion rate, which puts them at risk to blood borne infections. Strict vaccination policy as well as improving students’ awareness on the protective role of vaccination against NSI-associated infectious diseases are necessary.

The students seem uncertain what to do to reduce the risk of infection after NSI. Only third of them identified the need to wash the area with soap and water, which validates the fact that only over half of them washed the injured area immediately. One study showed nursing students carrying out other first aid measures such as squeezing the site or applying pressure similar to this study [26], which oppose recommended actions. Poor knowledge and practice in first aid measures may put students at risk to infection.

No significant difference in knowledge is noted with regard to students’ gender and year level; whereas in one study, knowledge is associated with year level but not in gender [18]. BSN bridging students has significantly higher knowledge compared to the regular BSN students similar to previous work [20]. Bridging students already has substantial years of clinical experience working as staff nurses after their Diploma education and may have attended staff development trainings on NSI. Consequently, students who received previous education about NSI had significantly higher total knowledge score that those who did not [29]. Indeed, the lack of knowledge and training on policies, protocols and guidelines on NSI leads to extremely higher risk of occurrence [31]. The findings on students’ knowledge of NSI prevention altogether highlight the relevance of instituting educational and training programs to enhance nursing students’ knowledge on NSI and to correct misperceptions.

### Limitations of the study

The finding of this study is valuable as it adds to the existing body of knowledge on NSI. Nevertheless, caution must be exercised in interpreting the results due to some limitations. First, the study was conducted in a single university in Oman, which limits the generalizability of the findings. Also, the knowledge and experiences on NSI of the students included in the study may not be the same with other students who did not take part in the study. Future studies may include other nursing schools with a bigger sample size for a more conclusive outcome. Lastly, response bias cannot be circumvented with self-reported online data collection.

## Conclusion

This study demonstrate that nurse students had moderate level of knowledge about NSI prevention measures and lack the safe infection control practice in many facets. These findings have implications on the current clinical practices and might require a collaborative effort of nursing administrators from the academe and clinical areas to develop effective strategies not only to lower but also to eliminate the occurrence of NSI. As for improving the knowledge levels of nursing students, we recommend revisiting the nursing curriculum, developing long-term educational and training programs, tightening the implementation of policies on infection control and NSI prevention such as needle recapping and simplifying reporting protocol, and ensuring the availability of educational courses to promote safer practices.

## Data Availability

The datasets generated and/or analysed during the current study are not publicly available due [restrictions by the Research and Ethics Committee in the College of Nursing at Sultan Qaboos University to protect the participants’ privacy] but are available from the corresponding author on reasonable request.

## Abbreviation

NSI: Needle stick injuries
